# The Elberfeld System

**Published:** 1888-04-28

**Authors:** 


### The Hospital

A Weekly Institutional Journal of

**Science, Medicine, Nursing, and Philanthropy.**

*For Hospitals, Asylums, Medical Practitioners, Students, Nurses, Families, Parishes, Congregations, and the Charitable Public.*

Vol. IV. No. 83.] [April 28, 1888.

### THE ELBERFELD SYSTEM.

What is the Elberfeld system? It is a particular method of helping poor people, devised and carried out by the German town of Elberfeld. Like other new and successful departures, it has attained a certain degree of notoriety, and now Berlin, Hamburg, and other well-known places are thinking it may also be of use to them. In Great Britain there is, among all sorts and conditions of men and women, a great deal of sympathy with the poor. If we only knew exactly what to do, there are among us innumerable willing hearts and hands to do it.

Mr. Ritchie, the President of the Local Government Board, has come to the help of citizens who feel deeply their responsibility to those who are less prosperous than themselves. Some little time ago he did the most practical thing possible: he sent capable and experienced men to see the Elberfeld system at work in its own home. What we should like to do would be to send influential and kind-hearted men and women from every parish in the kingdom to Elberfeld, in order that they too might find out by actual experience how the system works. The visitors selected by Mr. Ritchie were Mr. J. S. Davy, Mr. C. S. Loch (secretary to the Charity Organisation Society), and Mr. Hanewinkel (of the Liverpool Central Relief and Charity Organisation Society). Those gentlemen spent some time at Elberfeld, and thoroughly examined the system in all its details. They have now returned, and issued an official report, which, it may be hoped, will be largely circulated among all those who take a practical interest in the poor-law system and other economic questions.

As might have been anticipated, the Elberfeld system is marked by great simplicity. It is a system according to which the ratepayers join hands and help, instead of delegating every possible duty to paid officials. In this country officialism is rampant. Whenever a disagreeable duty has to be done, unless it be a duty which conscience declares cannot be lawfully shirked, the average Englishman would much rather pay another to do it than do it himself. It is probable that the same state of things existed at Elberfeld, and that finally the inefficient management and the unrelieved suffering and over-relieved knavery became intolerable. However that may be, Elberfeld invented a system of her own, which she has worked successfully, and which is spreading to other towns. Here is the system in brief: Elberfeld is a large manufacturing town of over one hundred thousand inhabitants, and contains a good many poor. For poor-law purposes it is divided into three hundred and sixty-four sections; each section therefore consisting of about three hundred people, more or less. Every fourteen sections constitute a district. Over each section of three hundred inhabitants is placed an almoner; and over each district, which has fourteen almoners, is appointed an overseer. All these officers are under the control of a central committee of nine, of which the mayor is *ex-officio* president, four members are town councillors, and the other four are ordinary citizens. The members of this central governing body are selected for their proved capacity in dealing with financial questions.

The system looks very orderly and complete on paper, but the practical man asks, Where do you get your three hundred and sixty-four almoners and your twenty-six overseers? Are they all paid officials; or, if they are not paid, have they nothing to do that they can afford to spend so much of their time in looking after the poor? They are not paid officials, and, as a matter of fact, they do spare the time. Their willingness may be a little helped by one of the Prussian municipal laws which enacts that every ratepayer is bound under penalties to undertake any unpaid office in the poor-law administration for which he may be chosen. There is no reason whatever why the separate municipalities in this country should not each enact a similar law. One distinguishing merit of this system is that it makes the ordinary citizen, whether he will or not, thoroughly familiar with the straits and sorrows of the poor, with the causes of distress, and with the various methods of relief. It recognises frankly that people living in communities and enjoying the protection, the social and intellectual intercourse and the other manifold advantages of civilised society, cannot have the gains of civilisation without recognising its obligations. Preachers are constantly reminding us that we are our brothers' keepers, and some of us admit, whilst others cynically deny the proposition. In Prussia they are more business-like and practical. Instead of the preacher vainly pleading the doctrine of brotherly care, the sterner tones of the law speak out and say: "You *must* take care of your poorer brother whether you will or not." When the doctrine of brotherhood comes with State authority converts are never wanting. The Elberfeld system deserves well, in that it refuses to permit the citizens to compound for their personal duty to their fellow-men by a money payment. The community as a community recognises the necessity for personal work as well as for contributions to the rates. Each individual of the community holds himself in readiness at the call of the municipality to take his share of intelligent and efficient work.

The duties of the almoners are extensive and precise. Every person needing relief makes application to the almoner of his own district. It is then the duty of the almoner to institute full and particular inquiries into all the circumstances of the case. He is also required to keep himself constantly informed so long as the applicant may continue to need relief. Every fortnight the fourteen almoners of each district meet under the presidency of the district overseer; the reports of each are then considered, and the minute-book prepared for the Central Committee. That committee also meets fortnightly on the day following the meetings of the almoners and overseers. The small size of the sections enables each almoner, without difficulty, to make himself thoroughly familiar with all the distress of his district, and with every exaggerating or favourably modifying circumstance connected with it. Being a citizen and not a paid official, he has no interest but to state the facts as plainly as may be, and to secure that the relief shall be such as is best suited to the necessities of each case. Relief is granted according to a fixed and uniform scale, which is so framed as to secure that only the minimum necessary for bare subsistence is supplied to the applicant and his family. Any small sums he may earn are considered, and deducted so as to bring his rate of relief to the standard minimum. In addition to money help, tools may be lent—such as sewing machines; and furniture also may be provided. One of the instructions of the almoner is that he is bound to use every possible effort to secure employment for those who may be in receipt of relief.

This is a simple, complete, and effective scheme of poor-law relief. That it would work well in this country is absolutely certain, because it would work well anywhere in the hands of people of average intelligence and conscience. Some such system is adopted by many ecclesiastical organisations, but it fails to a large extent because those organisations overlap one another in all directions, and are always more or less competing with each other for popular favour. The admixture of motives of that kind is fatal to any general success. Mr. C. S. Loch considers that the great difficulty in the way of organising charitable relief on an intelligent and effective system is that the benevolent world is so divided into "cliques, classes, and ecclesiastics." No doubt there is much truth in Mr. Loch's assertion, and he and his subordinates have probably often experienced the stinging power of angry and excited ecclesiastics of both sexes. But the adoption of a municipal system which should divide the parish into equal districts, and appoint to each district a parish almoner of its own, would prevent overlapping and put a stop to the mutual jealousies which demoralise the poor and waste the resources of the charitable.

At the present time it would be exceedingly difficult to obtain a sufficient number of almoners and overseers to carry out the Elberfeld system thoroughly. But there is no reason why the broad principles of the system should not be adopted and its details modified to suit our national character and peculiar circumstances. With us there are many people who have time at disposal, enough probably, at any rate, to provide at least the requisite overseers. The larger class of almoners might probably be to some extent recruited from the ranks of benevolent ladies. Ladies, it seems to us, are eminently fitted to act as almoners under suitable supervision. If it were attempted to pass a municipal law in any town, making it compulsory for every inhabitant to accept any unpaid office under the poor-law to which he might be appointed, there would probably be a very loud outcry. But there is hope that Liverpool will voluntarily undertake to try a system similar to, if not identical with, that of Elberfeld; and if the northern city should show a markedly successful result it may be safely anticipated that other large towns will rapidly follow. It is grievous to every public-spirited inhabitant of London to think that in such cases as this, the metropolis, which should naturally lead the way, is powerless. So huge is its bulk, and such is the condition of confusion worse confounded into which its government has gradually drifted, that its power of inception or of individual action is practically *nil.* There are, however, in England and Scotland many large and populous towns and cities each having its own perfectly independent municipal system. There is a fine opportunity for those important cities to march side by side with Liverpool in a peaceful revolution which may do much to satisfy the consciences of the benevolent and to unite in closer and more human bonds a society in which the elements of separation and dissolution are increasing at no halting pace.

